# Identifying priorities in methodological research using ICD-9-CM and ICD-10 administrative data: report from an international consortium

**DOI:** 10.1186/1472-6963-6-77

**Published:** 2006-06-15

**Authors:** Carolyn De Coster, Hude Quan, Alan Finlayson, Min Gao, Patricia Halfon, Karin H Humphries, Helen Johansen, Lisa M Lix, Jean-Christophe Luthi, Jin Ma, Patrick S Romano, Leslie Roos, Vijaya Sundararajan, Jack V Tu, Greg Webster, William A Ghali

**Affiliations:** 1 Department of Community Health Sciences, University of Calgary, Calgary, Canada; 2 Centre for Health and Policy Studies, University of Calgary, Calgary, Canada; 3 Information Services, Healthcare Information Group, Edinburgh, UK; 4 British Columbia Cardiac Registry, Vancouver, Canada; 5 Institut Universitaire de Médecine Sociale et Préventive, University of Lausanne, Switzerland; 6 Centre for Health Evaluation and Outcome Sciences, University of British Columbia, Vancouver, Canada; 7 Health Division, Statistics Canada, Ottawa, Canada; 8 Manitoba Centre for Health Policy, Department of Community Health Sciences, University of Manitoba, Winnipeg, Canada; 9 Public Health College, Second Shanghai Medical University, Shanghai, China; 10 University of California Davis School of Medicine, Davis, USA; 11 Department of Human Services, Melbourne, Australia; 12 Institute for Clinical Evaluative Sciences, Sunnybrook Health Sciences Centre, University of Toronto, Toronto, Canada; 13 Canadian Institute for Health Information, Toronto, Canada; 14 Department of Medicine, University of Calgary, Calgary, Canada

## Abstract

**Background:**

Health administrative data are frequently used for health services and population health research. Comparative research using these data has been facilitated by the use of a standard system for coding diagnoses, the International Classification of Diseases (ICD). Research using the data must deal with data quality and validity limitations which arise because the data are not created for research purposes. This paper presents a list of high-priority methodological areas for researchers using health administrative data.

**Methods:**

A group of researchers and users of health administrative data from Canada, the United States, Switzerland, Australia, China and the United Kingdom came together in June 2005 in Banff, Canada to discuss and identify high-priority methodological research areas. The generation of ideas for research focussed not only on matters relating to the use of administrative data in health services and population health research, but also on the challenges created in transitioning from ICD-9 to ICD-10. After the brain-storming session, voting took place to rank-order the suggested projects. Participants were asked to rate the importance of each project from 1 (low priority) to 10 (high priority). Average ranks were computed to prioritise the projects.

**Results:**

Thirteen potential areas of research were identified, some of which represented preparatory work rather than research *per se*. The three most highly ranked priorities were the documentation of data fields in each country's hospital administrative data (average score 8.4), the translation of patient safety indicators from ICD-9 to ICD-10 (average score 8.0), and the development and validation of algorithms to verify the logic and internal consistency of coding in hospital abstract data (average score 7.0).

**Conclusion:**

The group discussions resulted in a list of expert views on critical international priorities for future methodological research relating to health administrative data. The consortium's members welcome contacts from investigators involved in research using health administrative data, especially in cross-jurisdictional collaborative studies or in studies that illustrate the application of ICD-10.

## Background

Health administrative data are frequently used for health research in Canada and abroad. In the past two decades, such data have been widely employed by health services and population health researchers to study healthcare outcomes, effectiveness, appropriateness and utilization of healthcare services, and to investigate or monitor population health status and its determinants [1-11]. The varied and broad use of administrative data has been facilitated by important advantages of the data, including their accessibility, their wide geographic coverage and their relatively complete capture of contacts with the health system for a defined population [12,13].

The use of health administrative data in health services research has been enabled by some key characteristics, notably the use of a standard system for coding diagnoses, the International Classification of Diseases (ICD). Established by the World Health Organization in 1893 to categorise causes of death, this system adopts a standardised format to code diagnoses, thereby enabling longitudinal and comparative studies [14]. The ninth revision, ICD-9, was expanded in 1977 to ICD-9-CM (Clinical Modification) to enable more precision in diagnostic codes, together with the addition of surgical intervention codes. In 1992, the 10^th ^Revision of ICD (ICD-10) was introduced. ICD-10 has been used by many countries throughout the world for coding cause of death and for hospital diagnoses since 1994 [15-17]. It has been used for mortality data since 2000 in Canada, and provinces have adopted ICD-10 for coding hospital diagnoses in a phased approach, beginning in 2001.

One of the major advantages of ICD-10 is that it is far more detailed (there are a total of 12,420 codes in ICD-10 compared to 6,969 in ICD-9), permitting richer capture of clinical information. However, its implementation means that a number of established methodological tools applicable to ICD-9 or ICD-9-CM need to be redesigned for application in ICD-10. Another issue is that the structure of ICD-10 differs substantially from ICD-9. Furthermore, since each country licences the coding system individually from WHO and can create its own modifications, there may be more opportunity for discrepancies between countries. Finally, ICD-10 does not include procedure codes and so each country has developed its own coding system. The system used by Canada is the International Classification of Diseases, 10^th ^revision, Canadian version, Canadian Classification of Health Interventions (ICD-10-CA/CCI).

Clearly the implementation of ICD-10 offers many benefits while also raising significant challenges for the international health services and population health research communities. In addition, research using ICD administrative data must address other limitations, largely stemming from the fact that the data were created not for research but for other purposes. Data quality is a concern; errors in the data can stem from inaccurate or missing information in the patient record, from the failure to abstract relevant data, or from incorrect coding of the abstracted data. Another concern is that administrative data lack clinical details. Even when data quality is good, the diagnoses that are coded do not reflect the severity of disease, diagnostic findings are not coded, and clinical sequence is not available.

This paper describes the origins and first symposium of a new international group that has come together to discuss how to take advantage of these potential benefits, and to address the new and ongoing challenges associated with using administrative data in health services and population health research. International collaborative research on health services has many advantages. From the methodological perspective, such research allows investigators to develop analytic tools that are more robust and more generalisable. It also allows those tools to be adopted in a systematic and uniform manner across countries, thereby fostering international exchange of research data and findings. From the policy perspective, it helps us to understand the strengths and weaknesses of various healthcare systems, and identifies opportunities for improvement in those systems.

### The consortium

The consortium came together through a fortuitous set of circumstances. Australian researcher Vijaya Sundararajan contacted Canadian researchers William Ghali and Hude Quan because they were all doing similar work. While on sabbatical, William Ghali met Swiss researchers with similar interests: Patricia Halfon, Jean-Christophe Luthi and Bernard Burnand. These links led to two initial collaborative projects: new ICD-10 coding algorithms for two widely-used comorbidity measures, the Charlson index and the Elixhauser comorbidity categories [18].

Meanwhile the Canadian Institutes of Health Research (CIHR) announced a funding opportunity for workshops. A successful proposal by Ghali and Quan to the Institute for Health Services and Policy Research permitted a seminar and workshop held June 17 and 18, 2005 in Calgary and Banff, Alberta. The objectives of the workshop were to:

1) solidify collaborative relationships through a face-to-face meeting of researchers;

2) initiate dialogue around launching a set of collaborative research projects on methodological issues surrounding the use of administrative data; and

3) stage a symposium in parallel to the workshop meetings at which the invited researchers would present their work to interested attendees.

Additional invitees to the seminar and workshop included representatives from two stakeholder organizations (Canadian Institute for Health Information (CIHI), and Statistics Canada), five Canadian collaborators, and investigators from the United States, the United Kingdom, Australia, Switzerland and China. The list of invited participants was a convenience sample whose selection was based on two criteria: they were bona fide experts in this area and/or they were known to the organisers.

## Methods

The Seminar was held on the morning of June 17 at the Faculty of Medicine, University of Calgary. Members of the international consortium gave 11 presentations to an audience of approximately 100 people, with participants from not only Calgary, but also Edmonton, Vancouver and Ontario. The workshop presentations included descriptions of administrative data systems in Switzerland, Scotland and China, and the use of administrative data to measure comorbidities, chronic disease prevalence, quality of care and waiting times.

The research planning workshop followed on Saturday, June 18 in Banff. The atmosphere was informal and collaborative. The morning sessions covered such topics as the validity of administrative data, analysis of administrative data by Statistics Canada, premature mortality in Scotland and Europe, and opportunities for using CIHI data for research. The group then engaged in a focussed discussion around ideas for future collaborative research projects necessary to advance this field. The emphasis in this research planning discussion was on high-priority methodological areas in need of research that the consortium could undertake collectively in future work. Some of the areas identified represent preparatory work rather than research *per se*.

## Results

Thirteen potential areas of research were identified.

1. 'Meta-data' documentation of international administrative data: Every field in each country's hospital administrative data system would be defined and described. While not as exciting as more applied projects, a compilation of this nature would be necessary for international comparative studies, and would also serve to highlight identified problems or issues with the data from specific countries.

2. International cross-validation of new ICD-10 coding algorithms. ICD-10 versions of the Charlson and Elixhauser comorbidity indices have been developed, as mentioned previously. There has been some initial work comparing the results of the new Charlson coding algorithms across countries, but more work is necessary. ICD-10 coding algorithms need to be developed in other areas, for example chronic diseases, along with additional international comparisons

3. Patient safety indicators (PSI) translation: PSIs have been developed using ICD9-CM coding, under the auspices of the U.S. Agency for Healthcare Research and Quality, but corresponding ICD-10 codes for these indicators have not yet been developed. The PSIs are designed to screen for potentially preventable adverse effects of hospitals care. By translating the PSIs into ICD-10 and then validating this translation using data that have been independently coded according to both ICD-9-CM and ICD-10, researchers will be able to compare inpatient safety across national boundaries.

4. Learning curves: This effort would focus on the timing of uptake of ICD-10, and whether data validity assessments indicate the presence of a learning curve for coding. Canada, with its phased implementation in multiple provinces over several years, would be an ideal setting for this type of work.

5. Training standards for health record coders: It was discovered at the workshop that hospital abstract coders receive very different training from country to country. This project would explore those issues further with formal documentation of training requirements and practice guidelines for health record coders in various countries.

6. Chart-Database comparison studies: This would involve medical record reviews to determine the validity of hospital abstract data compared with the patient record across multiple countries. These are very expensive studies, especially if international comparisons are involved, but they would help researchers to characterise the importance of reporting and coding bias in international studies using administrative healthcare data.

7. Internal consistency algorithms: Algorithms can be developed to verify the logic of codes. For example, diabetic retinopathy should not occur in a patient who has never had a diagnosis of diabetes; prostatectomies cannot occur in females. Some work of this type has already been done in Switzerland and California. Different algorithms could be tested, refined, validated and then made available to others.

8. "True" gold standard: The purpose of this research would be to verify whether the trusted gold standard in observational health research, the patient's medical record, is in fact valid when compared to a 'truer' gold standard of information collected prospectively from patients and providers during a medical encounter. This research would require real-time patient assessments by independent clinicians who would observe all of the patient's interactions with physicians, as well as all of the discussions among the physicians involved in establishing and treating the patient's diagnosis. Comparisons would then be made between the independent assessment, the patient record, a nurse reviewer, and administrative data.

9. Travelling coders for comparative recoding: This research would require travelling coders who would recode previously coded records across countries to assess uniformity. By using a single team of travelling coders, researchers could estimate the nature and magnitude of international differences in coding practices.

10. Interventional studies to enhance coding quality: This research might include, for example, randomised controlled trials or pre-post studies to determine the effectiveness of educational or system interventions aimed at improving coding quality.

11. Value of diagnosis type coding: Some countries (or individual states or provinces) include a diagnosis-type code indicating whether each diagnosis is a comorbidity or a complication. Research in this area would focus on demonstrating the value of diagnosis-type codes, their validity, and the economic and human resources impact of implementation.

12. International comparisons of predictive model performance, as measured by the C (concordance)-statistic: It was determined from the group's presentations that C-statistic values differ across countries in comorbidity-based mortality predictions, but it is not understood why. The C-statistic is a measure of the discriminative accuracy of a logistic regression model [19,20]. The difference in C-statistic values may depend on the number of diagnosis fields available in abstracts, as well as the underlying coding validity and the epidemiology of disease in the population. Research in this area would aim to uncover the factors that contribute to the observed differences in model performance.

13. International scan of privacy considerations across countries and implications regarding permissible linkage activities: Discussions at the workshop revealed that there are considerable differences between countries in permissible data linkage activities, which have a great impact on the types of health services research that is possible.

After the brain-storming session, voting took place to rank-order the suggested projects. Participants were asked to rate the importance of each project from 1 (low importance), to 10 (high importance). Average ranks were computed to prioritise the projects (Table 1). While all projects were considered to be of at least moderate importance, several priorities emerged, in particular, research into international meta-data documentation and translation of patient safety indicators.

## Discussion

Objectives were achieved; the workshop was considered by all to be a big success and a memorable event. Valuable face-to-face contacts were made and the addition of outdoor activities on Sunday June 19 helped to solidify linkages between participants. The group discussions resulted in a list of expert views on critical international priorities for future methodological research relating to health administrative data. It must be acknowledged, however, that the list was limited by the experience and knowledge of the experts who attended the meeting and as such, it is certainly possible that the list omits key issues that others would consider to be important.

Since the symposium, work has continued. A paper is in preparation comparing three ICD-10 translations of the Charlson comorbidity index that were developed in Switzerland, Australia, and Canada. Within Canada, trends in the coding of Charlson comorbidities are being analyzed, assessing the impact and learning curve associated with the phased introduction of ICD-10. Preparatory dialogue is underway to plan the implementation of additional projects in the research areas outlined in the table.

### Knowledge exchange

The consortium is committed to the dissemination and sharing of knowledge with the broader health services and population health research communities. The PowerPoint presentations from the seminar are available on the website of the Centre for Health and Policy Studies, University of Calgary [21]. Useful websites which describe methodological tools, key concepts and operational definitions emanating in part from the work of consortium members include the Manitoba Centre for Health Policy's concept index [22], the Centre for Health and Policy Studies [21], the Institut Universitaire de Médecine Sociale et Préventive [23], AHRQ's quality indicators [24], and the Canadian Institute for Health Information [25].

The consortium's members welcome contacts from investigators involved in research using health administrative data, especially in cross-jurisdictional collaborative studies and/or in studies that illustrate the application of ICD-10. All attendees indicated commitment to carry forward the enthusiasm evident at this inaugural workshop, and hoped to hold future consortium meetings to advance the exciting and important work of this international group.

## Competing interests

The author(s) declare that they have no competing interests.

## Authors' contributions

HQ and WAG obtained the funding for the workshop, organised and facilitated it, and assisted in drafting the manuscript; CD drafted the manuscript; all participants were actively engaged in the workshop, contributed to the discussions and ratings about priority areas for future collaborative research, and participated in revising the paper and reviewing the final version for submission.

## Pre-publication history

The pre-publication history for this paper can be accessed here:



## Figures and Tables

**Table 1:** Ranking of research areas in order of importance for administrative data research

Project	Mean score	Number of raters	Priority Rank
Meta-Data documentation	8.4	10	1
Patient safety indicators translation	8.0	10	2
Internal consistency algorithms	7.0	10	3
Interventional studies to enhance coding quality	6.9	10	4
"True" gold standard	6.8	10	5
Travelling coders for comparative recoding	6.8	9	5
Training standards for health records coders	6.4	10	7
International comparisons of predictive model performance (C-statistic)	6.2	10	8
Value of diagnosis-type coding	6.0	9	9
Chart-Database comparison studies	5.9	10	10
International cross-validation of ICD-10 coding algorithms	5.8	10	11
International scan of privacy considerations	5.6	10	12
Learning curve in ICD-10 uptake	5.0	10	13
